# Valerenic acid ameliorates amphetamine-related neurotoxicity by improving hypothalamus *tyrosine hydroxylase* and *histamine-N-methyl transferase* enzymes

**DOI:** 10.1016/j.toxrep.2025.101936

**Published:** 2025-01-29

**Authors:** Khaled M.M. Koriem, Ammar H.A. Naiem

**Affiliations:** a Medical Physiology Department, Medical Research and Clinical Studies Institute, National Research Centre, Dokki, Giza, Egypt; b Department of Pharmacology and Toxicology, Faculty of Pharmacy, Helwan University, Egypt

**Keywords:** Valerenic acid, Methamphetamine, Serotonin, Tyrosine hydroxylase, Interleukin-1β

## Abstract

**Background:**

Narcolepsy, obesity, and attention deficit hyperactivity disorder are all treated with amphetamine (a central nervous system stimulant) while valerenic acid (VA) has a pharmacological effect in the central nervous system.

**Objectives:**

The purpose of this study was to ascertain whether VA is able to make amends for neurotoxicity by modifying hypothalamus expressions of the enzymes *tyrosine hydroxylase and histamine-N-methyl transferase* in rats orally administered with methamphetamine (METH).

**Methods:**

There were thirty-six male albino rats split up into six equal groups, Control, VA (5 mg/kg)-treated, and VA (10 mg/kg)-treated groups: For four weeks, normal rats received oral administration of 1 ml of distilled water, 5 mg/kg of VA, and 10 ml/kg of VA once daily. METH-treated, VA (5 mg/kg) prior to METH-treated, and VA (10 mg/kg) before METH-treated groups: normal rats were oral administrated with METH (2.5 mg/kg), 3 days/week for 3 weeks, where the last two groups were oral administrated daily during four weeks at 5 mg/kg and 10 mg/kg VA, starting one week prior to METH administration.

**Results:**

METH decreased superoxide dismutase, glutathione peroxidase, catalase, NADPH oxidase, interleukin-10, sucrose preference test, distance traveled test, and center square entries test, ATPase activity and the enzymes *tyrosine hydroxylase* and *histamine-N-methyl transferase* but increased malondialdehyde, conjugated dienes, oxidative index, serotonin, dopamine, norepinephrine, γ-aminobutyric acid, tumor necrosis factor-α, interleukin-1β, interleukin-6, nuclear factor kappa B levels, the center square duration test, tail suspension test, and forced swimming test. in the METH-treated animals' brain in contrast to the control group. After four weeks of oral administration of VA to METH-treated rats, all of these parameters returned to levels that were nearly control, indicating that a higher dose was more effective than a lower one.

**Conclusion:**

VA ameliorated METH-related neurotoxicity by improving hypothamalus expressions of the enzymes *tyrosine hydroxylase* and *histamine-N-methyl transferase*.

## Highlights

• Valerenic acid neutralized methamphetamine induced behavioral deficits.
• Valerenic acid amended hypothalamus sodium/potassium-ATPase activity.
• Valerenic acid restored hypothalamus *tyrosine hydroxylase* and *histamine-N-methyl transferase*.
• Valerenic acid neuroprotective due to restore antioxidants and abolish inflammation.

## Graphical Abstract

**Figure:**
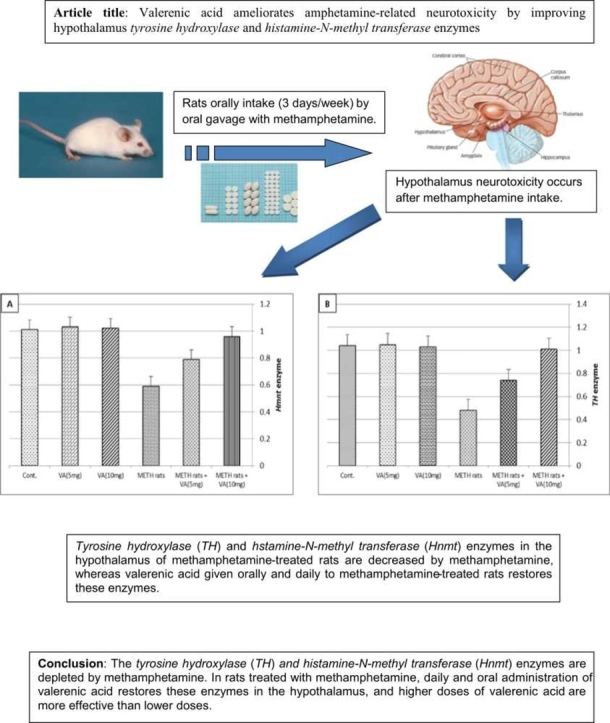

## 1 Introduction

Narcolepsy (a sleep condition), obesity, and attention deficit hyperactivity disorder (ADHD) are all treated with amphetamine (a central nervous system stimulant) [1], [2], [3]. Amphetamine is widely used and has characteristic toxidromes and potential for neurological injuries. It causes a euphoric high that builds in intensity. Rhabdomyolysis, ischemic and hemorrhagic strokes, seizures, and other movement disorders. Hallucinations, altered sensorium, skewed perception, and cognitive impairment are all displayed by amphetamine users [4]. Compared to healthy persons, amphetamine users reported increased subjective rates of impulsivity, and they also exhibited widespread prefrontal hyperconnectivity [5]. Higher increases in striatal dopamine release are linked to amphetamine administration. Neural disturbances caused by amphetamine in a number of extrastriatal areas, including the thalamus, insula, orbitofrontal cortex, and secondary somatosensory area [6]. Decision-making and person reaction are influenced by amphetamine abuse. It also altered brain integrity and response state, which indicating deficiencies in dopamine function [7]

The plant *Valeriana officinalis* is the source of valerenic acid (VA) and it is safe without any side effect [8]. *Valeriana officinalis*' plant belongs to Family: Valerianaceae and *Valeriana officinalis*' plant treats sleep disorders [9]. *Valeriana officinalis* plant is wide-spreading in Europe, Asia, and North America [10]. VA has an important pharmacological effect due to its impact on the neurons where VA acts as a γ-aminobutyric acid(A)-receptor modulator [11], [12]. Neuhaus et al. [13] proved that VA can infiltrates and passes the blood brain barrier in humans. Also, Hendriks et al. [14] stated that VA significantly reduced the amount of time that mice spent moving around and increased sleeping time in pentobarbital exposure. Moreover, VA has a considerable anxiolytic effect (Benke et al. [15] and Murphy et al. [16]). Furthermore, Becker et al. [17] demonstrated that VA cures people with anxiety and sleeplessness by shifting γ-aminobutyric acid-A receptors, it produces anxiolytic effect.

The purpose of this study is to assess how VA can reduce neurotoxicity in rats oral administrated with methamphetamine (METH) via altering the hypothalamus enzymes *tyrosine hydroxylase* and *histamine-N-methyl transferase*.

## 2 Materials and methods

### 2.1 Materials

Minapharm Pharmaceutical Company in Giza, Egypt, was the supplier of METH, while VA (99.8 % purity) was bought from ChromaDex Inc. in the United States. Authors bought lubrol, lucigenin (9, 9′-bis[N-methyl acridinium nitrate]), potassium phosphate monobasic (KH_2_PO_4_), and niacinamide-adenine dinucleotide phosphate (NADPH), from Sigma-Aldrich Co. in the US. All of the kits and reagents needed for the biochemical analysis were purchased by a local Bio-diagnostics Company (UK) branch in Egypt.

### 2.2 The study animals

Twelve-week-old male albino rats of the *Sprague Dawley* strain weighing 140–145 g were removed from the National Research Center's animal house colony in Egypt. They were housed in plastic cages, fed rat food, and had free access to tap water. The study was conducted in accordance with the National Regulations on the Animal Welfare of the National Research Center's Institutional Animal Ethical Committee (IAEC), and approval number 13051106 was given. Animals used in the study were treated and cared in accordance with NIH publication no. 85:23, revised in 1985.

### 2.3 Research plan

There was no prior research of VA oral dose on METH-treated rats therefore a dose-response curve of VA on METH-treated rats was done by choosing 10 doses of VA (0.5, 1.0, 1.5, 2.0, 2.5, 3.0, 3.5, 4.0, 4.5 & 5.0 mg/kg) were examined. Rats treated with METH (6 rats/group) received each of these doses orally for a week. Rats were scarified after a week in order to measure the neurotransmitters in the hypothalamus. The ideal dose noted in the neurotransmitters determination was 5 mg/kg VA.

According to the findings of the aforementioned study, 36 male albino rats were split up into 6 equal groups, each including 6 rats: (1) Control group: for four weeks, normal rats received 1 ml of distilled water orally once daily. (2) VA (5 mg/kg)-treated group: for four weeks, VA (5 mg/kg) diluted in one ml of distilled water was given orally once day to normal rats. (3) VA (10 mg/kg)-treated group: for four weeks, VA (10 mg/kg) diluted in one ml of distilled water was given orally once day to normal rats.(4) METH-treated group: For three weeks, normal rats were administrated 2.5 mg/kg of METH orally [18] once day, three days a week (Sunday, Tuesday, and Thursday), dissolved in 1 ml of distilled water. (5) VA (5 mg/kg) prior to METH-treated group: for four weeks, normal rats received a daily oral dose of 5 mg/kg of VA dissolved in 1 ml of distilled water. An hour later, the rats received 2.5 mg/kg of METH dissolved in 1 ml of distilled water, which was given orally for three days (Sunday, Tuesday, and Thursday) per week for three weeks, with VA oral administration occurring one week prior to METH oral administration. (6) VA(10 mg/kg) prior to METH-treated group: For four weeks, normal rats received a daily oral dose of 10 mg/kg of VA dissolved in 1 ml of distilled water. An hour later, the rats received 2.5 mg/kg of METH dissolved in 1 ml of distilled water, which was given orally for three days (Sunday, Tuesday, and Thursday) per week for three weeks, with VA oral administration occurring one week prior to METH oral administration.

For four weeks, oral gavage was used once daily to provide all of the previously indicated treatments. Every rat in the experiment was monitored for any unusual symptoms, including skin patches, convulsions, rat hair loss, and any fatalities.

### 2.4 Behavioral assessments

#### 2.4.1 Test of Sucrose Preference

This test was conducted using the Strekalova and Steinbuch [19] approach. This method involved keeping the rats in cages with two bottles of sucrose (1 % w/v) for 72 h in order to educate them to absorb sucrose. After then, one bottle was replaced with one that contains tap water. Both the amount of water and sucrose consumed were assessed in order to ascertain the sucrose preference.

#### 2.4.2 Test of forced swimming

Zhang et al. [20] method was used to find this test. The authors filled a plastic cylinder measuring 25 cm in diameter and 50 cm in height with water that was between 23 and 25 °C until it reached a predetermined height of 45 cm. Each rat spent five minutes immersed in this cylinder. After the rat taken from the water, it was left to dry. All the rats were put back in their cages. In order to calculate the immobility period, we measured the amount of time each rat spent floating in the water without exerting any effort to keep its head above the surface.

#### 2.4.3 Test of tail suspension

The methods suggested by Castagné et al. [22] and Belovicova et al. [21] were used to compute the test results. For five minutes, the animals were kept 58 cm above the ground and suspended separately from their tails. The rat became immobilized following the struggle phase, and each rat's immobility period was recorded.

#### 2.4.4 Test on the open field

The open field test was conducted using the Zhang et al. [20] technique, which consists of the center square duration, center square entries, and distance traveled tests. A cage that was extra-large, measuring 75 cm by 75 cm by 40 cm, was composed of 25 squares. Each animal was assessed independently after being allowed to explore the area for five minutes. Calculations for the number of crossings, rearing, and center square entrance timings were part of every session.

### 2.5 Preparation of hypothalamus tissue

The rats' heads were obtained and dissected following the final dose of each treatment. The brain tissue's hypothalamus was taken out and submerged in saline solution. The method for isolating the hypothalamus by Palkovits [23] was used. The total hypothalamus, including the supraoptic, tuberal, and mammillary regions, was examined in this study.

### 2.6 Biochemical assessments

#### 2.6.1 Hypothalamus antioxidants determination

The activity of superoxide dismutase (SOD) was measured using the Suttle [24] technique. The method developed by Pagalia and Valentine [25] was used to measure the activity of glutathione peroxidase (GPx). Authors measured the catalase (CAT) activity using the Aebi [26] technique. As a sign of lipid peroxidation, malondialdehyde (MDA) was measured using the Okhawa et al. [27] technique. Following the kit guidelines, spectrophotometry equipment was used to identify each of the aforementioned antioxidants. The conjugated dienes (CD) were obtained using the Kogure et al. [28] technique. The absorbance ratio A_233_/A_215_ [oxidative index (OI)] was computed following CD was measured using a spectrophotometer [29], [30]. Superoxide radical (O_2_^-^) production in NADPH oxidase activity was measured using a chemiluminescence technique [31]. Using the earlier method [32], NADPH oxidase activity was measured.

#### 2.6.2 Hypothalamus neurotransmitters determination

Serotonin (5-HT) level was determined using the Kitagawa [33] technique. The dopamine (DA) level was detected using the Guo et al., [34] technique. Using the method developed by Kapoor and Chalmers [35], the norepinephrine (NE) level was calculated. The concentration of γ-aminobutyric acid (GABA) was estimated using the Sciotti et al., [36] technique. Acetylcholinestrase (AchE) activity was detected using the Ellman et al. [37] technique. For each of the neurotransmitters described above, ELISA kits were utilized, and the kit's instructions were followed.

#### 2.6.3 Identification of inflammatory markers in the hypothalamus

The tumor necrosis factor-α (TNF-α) was identified using the Matalka et al., [38] technique. The amount of interleukin-1β (IL-1β) was measured using the DeCicco et al., [39] method. Calculations of interleukin-6 (IL-6) and interleukin-10 (IL-10) were performed using the Stelmasiak et al., [40] methodology. All of the previously described inflammatory markers were calculated using ELISA kits, which were utilized in accordance with the kit instructions.

### 2.7 Nuclear factor kappa B of the hypothalamus

Nuclear factor kappa B (NF-kB) in the hypothalamus was detected using the Eliza kit. The process was as follows: NF-kB from the hypothalamus was added to a monoclonal antibody enzyme well that had been pre-coated with rat NF-kB monoclonal antibody and incubated. NF-kB antibodies labeled with biotin were then added and combined with Streptavidin-HRP to form an immune complex. The uncombined enzyme was then removed by further incubation and washing. The absorbance was measured at 450 nm. The amount of NF-kB in the brain hypothalamus is directly correlated with the color that is produced [41].

### 2.8 Measurement of sodium/potassium-ATPase in the hypothalamus

Using the earlier method [42], the sodium/potassium ATPase activity was assessed. For this procedure, the primary components of the solution were ATP disodium salt (3 mM), KCl (20 mM), MgCl_2_ (5 mM), Tris HCl (50 mM, pH 7.4), and NaCl (80 mM). Sodium/potassium ATPase activity was then assessed. The reaction was started by adding the previously described solution, and it was then incubated with 50 ml of hypothalamus homogenate for 10 min at 37 °C. 50 ml of trichloroacetic acid was then used to halt the process. After that, the mixture was centrifuged for more five minutes at 3000 rpm. After that, 1 ml of the supernatant was taken out and put into a 250 ml solution that contained ammonium molybdate, trichloroacetic acid, and ascorbic acid. A spectrophotometer established at 680 nm to measure the obtained color.

### 2.9 Detection of the expression of the hypothalamic enzymes tyrosine hydroxylase (TH) and histamine-N-methyl transferase (Hnmt) by real-time PCR

The hypothalamus was treated with an Invitrogen® Pure to extract RNA.Link® RNA Mini Kit (Cat. No. 12183018A). The RNA-to-cDNATM Kit (Applied Biosystems kit reagents, Biorad Company), which converts cDNA to RNA, is the main technology used in this study. The primer (R_Th_1_SG) activates tyrosine hydroxylase. Histamine N-methyl transferase is activated by the primer Rn_Hnmt_1_SG, while glyceraldehyde 3-phosphate dehydrogenase (GAPDH) is activated by the primer Rn_GAPDH_1_SG. All these primers listed above were purchased from the Qiagen Corporation. Using 2-CT, the mRNA for the GAPDH gene was detected [43].

### 2.10 Statistical analysis

The data was presented in tables and figures as mean ± standard error mean (SEM).In this study, the gaussian distribution was used. With SPSS 13, a one-way analysis of variance (ANOVA) test was performed. *P*-values ≤ 0.05 were considered significant in *post-hoc* analysis using the fisher least significant difference (FLSD) test across all treatment groups.

## 3 Results

### 3.1 Animal behavior

**Fig. 1:**
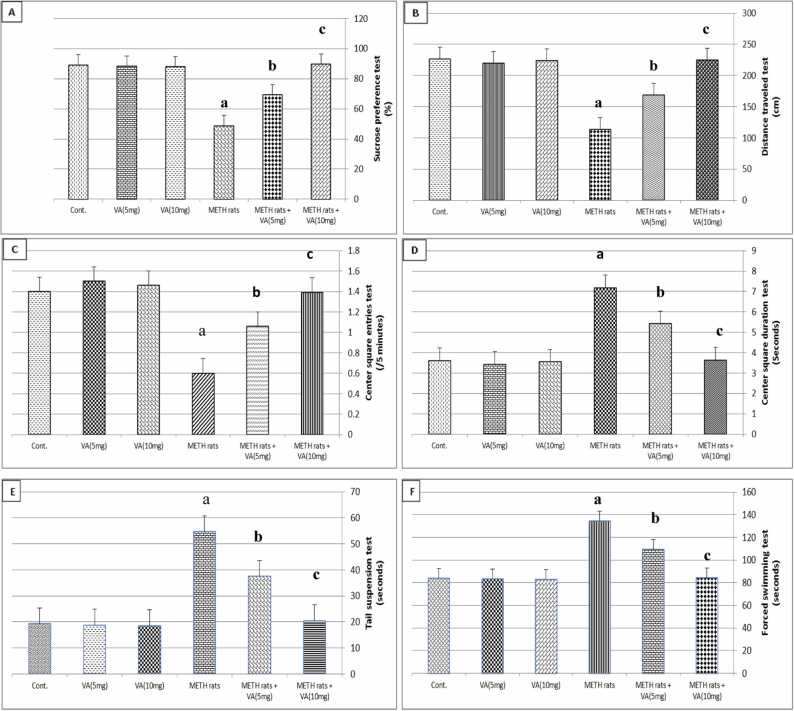
Effect of valerenic acid on behavioral tests of METH-treated rats. Number of animals= 6 rats/group. Data are represented as mean ± SEM. Cont.: Control. VA: Valerenic acid. METH: Metamphetamine. ^a^Highly significant change (*P* ≤ 0.01) compared to control. ^b^significant change (*P* ≤ 0.05) compared to METH-treated rats. ^c^Highly significant change (*P* ≤ 0.01) compared to METH-treated rats. Cont.: Control. VA: Valerenic acid. METH rats: Metamphetamine treated rats. METH rats+VA (5 mg): Metamphetamine-treated rats oral administraed with 5 mg/kg valerenic acid. METH rats+VA (10 mg): Metamphetamine-treated rats oral administraed with 10 mg/kg valerenic acid.

Fig. 1 shows the effect of VA on behavior tests in rats administered METH. In the center square entries, distance traveled test, and sucrose preference test, it is clear that METH caused a highly significant decrease (*P* ≤ 0.01) in comparison to the control group. A significantly significant increase (*P* < 0.01) in the center square duration, tail suspension test, and forced swimming test as compared to the control group. The aforementioned behavior tests (centre square entries test, sucrose preference test, centre square duration test, distance travelled test, forced swimming test and tail suspension test) in METH-treated rats were also pushed to be closer to the control values when administered orally with VA. The higher dose of VA was more effective than the lower dose in METH-treated group. Oral VA treatment (5 mg/kg and 10 mg/kg) did not, however, alter the behavior of normal rats in behavior tests.

### 3.2 Neurotransmitters in the hypothalamus

Table 1 displays how METH affects the levels of neurotransmitters (5-HT, DA, NE, and GABA) in the hypothalamus. When comparing the METH-treated rats to the control group, it is evident that METH elevated these neurotransmitters (*P* ≤ 0.01). Following oral administration of both doses of VA, where the action of VA was dose dependent, the aforementioned neurotransmitters were pushed to be around the control values in rats treated with METH. Additionally, the oral administration of VA to normal rats in this study did not alter any of the neurotransmitters.

**Table 1:** Effect of valerenic acid on neurotransmitters levels in hypothalamus of METH-treated rats.

**Parameters**	**Control**	**VA (5 mg/kg)**	**VA (10 mg/kg)**	**METH rats**	**METH rats + VA (5 mg/kg)**	**METH rats + VA (10 mg/kg)**
**Hypoth 5-HT (ng/g tissue)**	162 ± 5.01	164 ± 4.72	160 ± 4.92	250.1 ± 4.65^a^	205 ± 6.13^b^	160 ± 5.29^c^
**Hypoth DA (ng/g tissue)**	1120 ± 20	1118 ± 17	1122 ± 23	1648 ± 15^a^	1382 ± 17^b^	1118 ± 19^c^
**Hypoth NE (ng/g tissue)**	180 ± 6.12	182 ± 5.87	179 ± 7.04	265 ± 4.76^a^	221 ± 6.08^b^	179 ± 6.51^c^
**Hypoth GABA (µmol/g tissue)**	0.51 ± 0.06	0.49 ± 0.05	0.50 ± 0.07	0.75 ± 0.04^a^	0.61 ± 0.05^b^	0.43 ± 0.07^c^
**Hyp AchE activity (mmol/min/mg protein)**	3.6 ± 0.42	3.5 ± 0.43	3.7 ± 0.41	1.8 ± 0.39^a^	2.9 ± 0.42^b^	3.4 ± 0.42^c^

Number of animals= 6 rats/group. Data are represented as mean ± SEM. VA: Valerenic acid. METH: Metamphetamine. Hypoth: Hypothalamus 5-HT: Serotonin. DA: Dopamine. NE: Norepinephrine. GABA: γ-amino butyric acid. AchE activity: acetylcholinesterase activity.

a Highly significant change (*P* ≤ 0.01) compared to control.

b significant change (*P* ≤ 0.05) compared to METH-treated rats.

c Highly significant change (*P* ≤ 0.01) compared to METH-treated rats.

### 3.3 Antioxidants in the hypothalamus

Table 2 displays the impact of VA on antioxidant levels in the hypothalamus of rats administrated METH. According to the table, METH considerably (*P* ≤ 0.01) reduced the activities of NADPH oxidase, GPx, CAT, and SOD when compared to the control group. Comparing the OI, CD, and MDA to the control group revealed significantly significant increases (*P* ≤ 0.01) in METH-treated group with respect to the control rats. Oral administration of VA drove antioxidant tests for SOD, GPx, CAT activities, NADPH oxidase activity, MDA, CD, and OI to approach the control levels in comparison to METH-treated rats, where VA at higher dose was more effective than VA at lower dose. But when given orally to normal rats, VA did not alter the antioxidant levels in the hypothalamus.

**Table 2:** Effect of valerenic acid on antioxidants in hypothalamus of METH-treated rats.

**Parameters**	**Control**	**VA (5 mg/kg)**	**VA (10 mg/kg)**	**METH rats**	**METH rats + VA (5 mg/kg)**	**METH rats + VA (10 mg/kg)**
**Hypoth SOD (U/g tissue)**	3250 ± 60	3240 ± 50	3245 ± 55	1170 ± 40^a^	2200 ± 50^b^	3220 ± 70^c^
**Hypoth GPx (U/g tissue)**	785 ± 19	780 ± 24	785 ± 21	365 ± 18^a^	570 ± 16^b^	770 ± 21^c^
**Hypoth CAT (µmol H**_**2**_**O**_**2**_**/ min/mg tissue)**	0.18 ± 0.05	0.19 ± 0.04	0.17 ± 0.06	0.09 ± 0.03^a^	0.13 ± 0.06^b^	0.16 ± 0.05^c^
**Hypoth MDA (µmol/g tissue)**	8.52 ± 0.60	8.49 ± 0.72	8.50 ± 0.69	19.38 ± 0.54^a^	14.61 ± 0.83^b^	9.58 ± 0.90^c^
**NADPH oxidase activity (mg/mg protein x 10**^**5**^**)**	12.3 ± 1.18	12.5 ± 1.54	12.4 ± 1.37	7.6 ± 1.31^a^	9.6 ± 1.25^b^	12.1 ± 1.26^c^
**Hypoth CD (μmol/g tissue)**	1.45 ± 0.19	1.42 ± 0.17	1.43 ± 0.15	2.13 ± 0.14^a^	1.80 ± 0.19^b^	1.47 ± 0.17^c^
**Hypoth OI (A**_**233**_**/A**_**215**_**ratio)**	0.49 ± 0.03	0.47 ± 0.05	0.50 ± 0.03	0.82 ± 0.04^a^	0.67 ± 0.04^b^	0.51 ± 0.03^c^

Number of animals= 6 rats/group. Data are represented as mean ± SEM. METH: Metamphetamine. VA: Valerenic acid.

Hypoth: Hypothalamus SOD: Superoxide dismutase. GPx: Glutathione peroxidase. CAT: Catalase. CD: Conjugated dienes. OI: Oxidative index.

a Highly significant change (*P* ≤ 0.01) compared to control.

b significant change (*P* ≤ 0.05) compared to METH-treated rats.

c Highly significant change (*P* ≤ 0.01) compared to METH-treated rats.

### 3.4 The inflammatory markers of the hypothalamus

Table 3 shows how VA affected inflammatory markers in the rat hypothalamus following METH administration. It can be determined that METH led to a significant rise (*P* ≤ 0.01) in IL-1β, IL-6, and TNF-α levels in comparison to control values. Rats treated with METH showed significantly lower levels of IL-10 (*P* ≤ 0.01) than the control group. Furthermore, oral VA treatment to METH-treated rats caused these inflammatory markers to approach control levels, with higher dose of VA being more effective than the lower dose. Additionally, in the experiment, normal rats given oral VA showed no change in any of the inflammatory markers.

**Table 3:** Effect of valerenic acid on inflammatory markers in hypothalamus of METH-treated rats.

**Parameters**	**Control**	**VA (5 mg/kg)**	**VA (10 mg/kg)**	**METH rats**	**METH rats + VA (5 mg/kg)**	**METH rats + VA (10 mg/kg)**
**Hypoth TNF-α (ng/g tissue)**	46.8 ± 2.71	45.9 ± 2.68	47.3 ± 2.59	85.2 ± 2.59^a^	65.3 ± 2.64^b^	45.6 ± 2.48^c^
**Hypoth IL−1β (ng/g tissue)**	6.5 ± 0.4	6.4 ± 0.7	6.6 ± 0.5	13.1 ± 0.3^a^	9.7 ± 0.6^b^	6.3 ± 0.5^c^
**Hypoth IL−6 (pg/g tissue)**	54.6 ± 5.7	55.2 ± 6.1	53.8 ± 4.9	107 ± 4.5^a^	81.5 ± 7.2^b^	56.2 ± 6.3^c^
**Hypoth IL−10 (pg/g tissue)**	36.4 ± 3.9	34.8 ± 3.7	35.7 ± 4.0	17.5 ± 2.3^a^	26.5 ± 3.4^b^	33.9 ± 4.1^c^

Number of animals= 6 rats/group. Data are represented as mean ± SEM. VA: Valerenic acid. METH: Metamphetamine. Hypoth: Hypothalamus. TNF-α: Tumor necrosis factor-α. IL-1β: Interleukin-1β. IL-6: Interleukin-6. IL-10: Interleukin-10.

a Highly significant change (*P* ≤ 0.01) compared to control.

b significant change (*P* ≤ 0.05) compared to METH-treated rats.

c Highly significant change (*P* ≤ 0.01) compared to METH-treated rats.

### 3.5 NF-kB and Na^+^/K^+^-ATPase in the hypothalamus

**Fig. 2:**
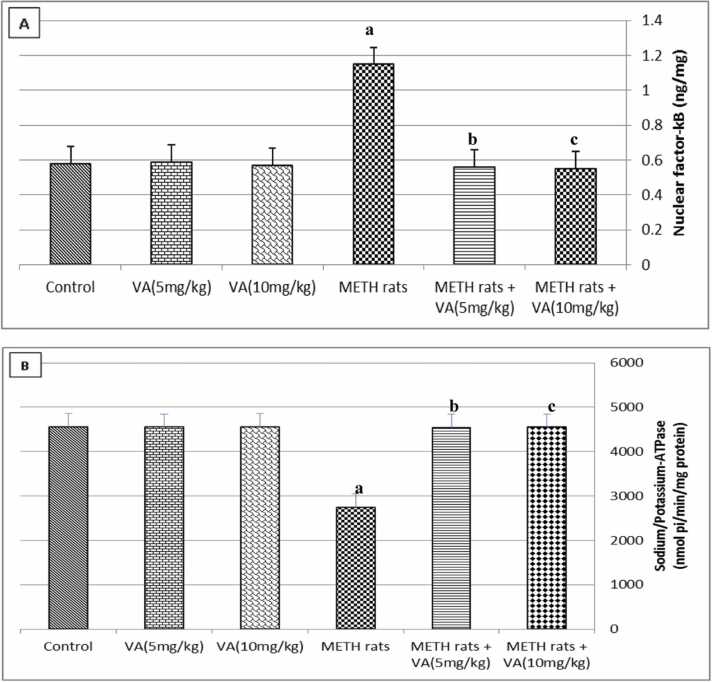
Effect of valerenic acid on nuclear factor kappa B and sodium/potassium-ATPase activity in hypothalamus of METH-treated rats. Fig. 2 reveals (A) hypothalamus nuclear factor-kappa B (ng/mg) and (B) hypothalamus sodium/potassium-ATPase activity (nmol pi/min/mg protein). Number of animals= 6 rats/group. Data are represented as mean ± SEM. VA: Valerenic acid. METH: Metamphetamine. METH rats + VA (5 mg): Metamphetamine-treated rats oral administraed with 5 mg/kg valerenic acid. METH rats + VA (10 mg): Metamphetamine-treated rats oral administraed with 10 mg/kg valerenic acid. ^a^Highly significant change (*P* ≤ 0.01) compared to control. ^b^significant change (*P* ≤ 0.05) compared to METH-treated rats. ^c^Highly significant change (*P* ≤ 0.01) compared to METH-treated rats.

Fig. 2 illustrates the impact of VA on Na^+^/K^+^-ATPase and NF-kB levels in the hypothalamus of the METH-treated group. This figure clearly shows that, in comparison to the control group, METH considerably enhanced NF-kB value but decreased Na^+^/K^+^-ATPase level (*P* ≤ 0.01). However, when oral VA was administered to METH-treated rats, the effects of VA were dose-dependent and NF-kB level and Na^+^/K^+^-ATPase value returned to levels that were nearly equal to control values. Furthermore, throughout the study, normal rats received oral VA treatment without seeing any changes in Na^+^/K^+^-ATPase or NF-kB values.

### 3.6 The enzymes tyrosine hydroxylase (TH) and histamine-N-methyl transferase (Hnmt) in the hypothalamus

**Fig. 3:**
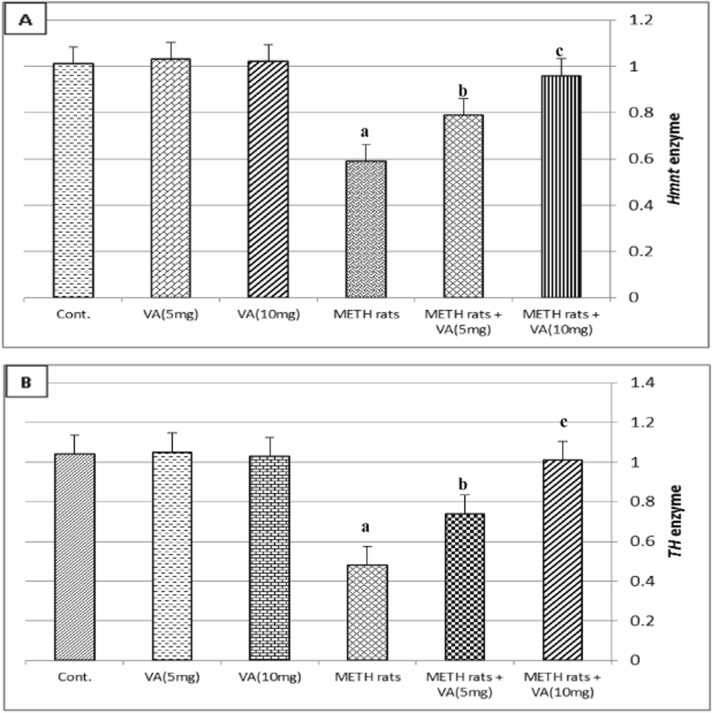
Effect of valerenic acid on fold change of histamine-N-methyl transferase (*Hnmt*) and tyrosine hydroxylase (*TH*) enzymes expressions in hypothalamus of METH-treated rats. Fig. 3 exhibits (A) hypothalamus histamine-N-methyl transferase (*Hnmt*) enzyme expression and (B) hypothalamus tyrosine hydroxylase (*TH*) enzyme expression. Number of animals= 6 rats/group. Data are represented as mean ± SEM. METH: Metamphetamine. VA: Valerenic acid. ^a^Highly significant change (*P* ≤ 0.01) compared to control. ^b^significant change (*P* ≤ 0.05) compared to METH-treated rats. ^c^Highly significant change (*P* ≤ 0.01) compared to METH-treated rats. Cont.: Control. VA: Valerenic acid. METH rats: Metamphetamine treated rats. METH rats+VA(5 mg): Metamphetamine-treated rats oral administraed with 5 mg/kg valerenic acid. METH rats+VA(10 mg): Metamphetamine-treated rats oral administraed with 10 mg/kg valerenic acid.

Fig. 3 illustrates how VA affects the fold change in the *TH* and *Hnmt* enzyme expressions in the rats' hypothalamus after receiving METH. When compared to the control group, METH significantly (*P* ≤ 0.01) decreased the levels of the *TH* and *Hnmt* enzymes in the rats treated with METH. Furthermore, giving VA orally to rats treated with METH causes the previously mentioned *TH* and *Hnmt* enzymes to approach control levels, where higher doses of VA were more beneficial than lower doses. Furthermore, oral VA treatment did not alter the levels of the *TH* and *Hnmt* enzymes in the hypothalamus in normal rats.

## 4 Discussion

The group that received METH showed a decrease in the sucrose preference test, distance traveled test, and center square entries test, but an increase in the center square duration test, tail suspension test, and forced swimming test. These results are consistent with those of several studies, like Hellem et al. [44], found female METH have higher depression than male METH users. Additionally, Keshavarzi et al. [45] reported METH reduces motor activity and cognitive function. METH causes worry and sadness. according to Yang et al. [46]. Also, Fu and Wang [47] observed amphetamine induces anxiety, depression, mania, and cognitive disorders. On the other hand, when METH-treated rats were given VA orally, their behaviors returned to a state that was almost the same as when they were in the control group; a higher dose of VA was more effective than a lower dose. These findings highlight VA's neuroprotective potentials. This result aligns with many studies, such as Ortiz et al. [48] found that VA abolished the decrease of brain excitatory transmission in the central depression. Also, VA improves the drug-metabolizing enzymes cytochrome P450 2D6 pathway of metabolism [49].

METH caused oxidative stress, metabolic impairment, and inflammation [50], [51], [52], [53]. The antioxidant enzyme activities in the rats' hypothalamus were increased when VA was administered orally to METH-treated rats. These findings are supported by other earlier studies, such as Kara et al. [54], which found that VA enhances redox homeostasis and safeguards against harmful xenobiotics. As a result, VA exerts ameliorative effects in oxidative stress because VA has strong antioxidant properties that are beneficial for shielding cells. Additionally, Wu et al. [55] showed VA has a strong antioxidant effect that inhibits important enzyme activities that are linked to many disorders, including type 2 diabetes, hypertension, and obesity. Moreover, Simsek et al., [56] showed the antioxidant potential of VA ingredient of epiphytic leafy liverwort. Furthermore, Dugaheh et al., [57] reported that VA is responsible for antioxidant activities of *Nardostachys jatamansi*, *Valeriana sisymbriifolia*, and *Valeriana officinalis*' plants.

In the hypothalamus, where METH is associated with mental and behavioral disorders [58] so METH increases 5-HT and DA transmission [59]. METH increased also levels of all other neurotransmitters such as NE and GABA while decreased AchE level. Oral VA treatment altered the neurotransmitters to nearby the control. Many studies, including Jung et al. [60], which demonstrated that VA regulates NE and 5-HT levels in the brain, support this conclusion. Additionally, Khom et al. [11], [12] demonstrated that VA directed to γ-aminobutyric acid(A) receptors and it consequently has anxiolytic action in vivo. Furthermore, Vidal-Cant et al. [61] reported that VA creates antinociception receptors at regions that are peripheral and spinal. Moreover, Trauner et al. [62] found Valerian extracts controls γ-aminobutyric acid(A) receptors. VA demonstrated high receptor activation where VA is utilized as an anti-inflammatory agent.

METH increases inflammatory cytokines to increase [63], which in turn causes inflammation and anxiety [64] consequently jumped NF-kB level. According to Jacobo-Herrera et al., [65], METH jumped NF-kB level and inflammation occurs but when METH-treated rats treated with VA orally, these inflammatory markers in the hypothalamus reduced to close normal levels. This finding relates to VA's ability to prevent the release of proinflammatory molecules such oncolukin-8 and TNF-α [66]. Additionally, VA significantly improved inflammatory tests, according to Nam et al., [67]. Consequently, VA decreases serum corticosterone levels, promotes cell proliferation and neuroblast differentiation, and enhances cognitive performance.and peroxidation of lipids.

METH also produced a drop in ATPase level because of the imbalance in the oxidative state in the hypothalamus of rats treated with METH. This occurred because METH caused oxidative stress, which led to the depletion of antioxidant enzymes [68]. This result is connected to the fact that oral administration of VA increased oxygen consumption while reduced glucose secretion, which is why the ATPase activity in the hypothalamus of METH-treated rats returned to normal after VA was administered [69], [70], [71]. VA also activates the phosphoenolpyruvate carboxylase cycle and plasma membrane-ATPase, which results in the restoration of ATPase value in the hypothalamus of rats given VA orally after receiving METH [72]. Finally, sodium/potassium-ATPase activity increased after oxidative stress balance occurs by VA oral intake [54].

*TH* and *Hnmt* enzymes overexpression declined in the hypothalamus of rats given METH. Histamine is a neurotransmitter that controls numerous physiological systems. Histamine depletion results in a variety of neurological illnesses, including Parkinson's disease, depression, and sleep difficulties. The brain expresses the histamine-metabolizing enzyme *Hnmt*. It controls catecholamines production, the shortage results in a decrease in their concentration. An key enzyme for the removal of histamine is *Hnmt* [73], [74]. In METH-treated rats, hypothalamus *TH* and *Hnmt* genes were suppressed. However, because VA can lower neuro-inflammation and the production of hydrogen peroxide in neurons, VA is able to reverse the effects of METH on *TH* and *Hnmt* enzymes levels in METH-treated rats [54], [66], [67].

## 5 Conclusion

METH induced neurotoxicity and this effect is due to METH increased neurotransmitters and inflammatory markers while decreased antioxidants, ATPase value, and *TH* and *Hnmt* enzymes in METH-treated rats. VA oral administration has the ability to amend hypothalamus *Hnmt* and *TH* enzymes and consequently improved behavioral deficits in METH-treated rats. This result is linked to VA's ability to reduce neuro-inflammation and the generation of hydrogen peroxide. The influence of VA on METH-treated persons will be examined in the clinical study that will be carried-out, and if VA is able to reduce METH-related symptoms in those people, the results will be good.

## Abbreviations

VA, Valerenic acid; METH, Methamphetamine; NADPH, Niacinamide-adenine dinucleotide phosphate; SOD, Superoxide dismutase; GPx, Glutathione peroxidase; CAT, Catalase; MDA, Malondialdehyde; CD, Conjugated dienes; OI, Oxidative index; 5-HT, Serotonine; DA, Dopamine; NE, Norepinephrine; GABA, γ-aminobutyric acid; AchE, Acetylcholinestrase; TNF-α, Tumor necrosis factor-α; IL-1β, Interleukin-1β; IL-6, Interleukin-6; IL-10, Interleukin-10; NF-Kb, Nuclear factor kappa B; *TH*, *tyrosine hydroxylase*; *Hnmt*, *Histamine-N-methyl transferase*.

## Funding

This research received no external funding.

## Declaration of Competing Interest

The authors declare that they have no known competing financial interests or personal relationships that could have appeared to influence the work reported in this paper.

## Data Availability

Data will be made available on request.
